# Advances of liposomal mediated nanocarriers for the treatment of dermatophyte infections

**DOI:** 10.1016/j.heliyon.2023.e18960

**Published:** 2023-08-05

**Authors:** Seyed Amin Ayatollahi Mousavi, Abnoos Mokhtari, Mahmood Barani, Alireza Izadi, Alireza Amirbeigi, Narges Ajalli, Azam Amanizadeh, Sanaz Hadizadeh

**Affiliations:** a Department of Medical Parasitology and Mycology, Faculty of Medicine, Kerman University of Medical Sciences, Kerman, Iran; b Medical Mycology and Bacteriology Research Center, Kerman University of Medical Sciences, Kerman, Iran; c Endocrinology and Metabolism Research Center, Institute of Basic and Clinical Physiology Science, Kerman University of Medical Sciences, Kerman, Iran; d Physiology Research Center, Kerman University of Medical Sciences, Kerman, Iran; e Department of General Surgery, School of Medicine, Kerman University of Medical Sciences, Kerman, Iran; f Department of Chemical Engineering, Faculty of Engineering, University of Tehran, Tehran, Iran

**Keywords:** Liposome, Nanotechnology, Dermatophyte, Topical, Fungi

## Abstract

Due to the adverse effects associated with long-term administration of antifungal drugs used for treating dermatophytic lesions like tinea unguium, there is a critical need for novel antifungal therapies that exhibit improved absorption and minimal adverse effects. Nanoformulations offer a promising solution in this regard. Topical formulations may penetrate the upper layers of the skin, such as the stratum corneum, and release an appropriate amount of drugs in therapeutic quantities. Liposomes, particularly nanosized ones, used as topical medication delivery systems for the skin, may have various roles depending on their size, lipid and cholesterol content, ingredient percentage, lamellarity, and surface charge. Liposomes can enhance permeability through the stratum corneum, minimize systemic effects due to their localizing properties, and overcome various challenges in cutaneous drug delivery. Antifungal medications encapsulated in liposomes, including fluconazole, ketoconazole, croconazole, econazole, terbinafine hydrochloride, tolnaftate, and miconazole, have demonstrated improved skin penetration and localization. This review discusses the traditional treatment of dermatophytes and liposomal formulations. Additionally, promising liposomal formulations that may soon be available in the market are introduced. The objective of this review is to provide a comprehensive understanding of dermatophyte infections and the role of liposomes in enhancing treatment.

## Graphical abstract

**Figure:**
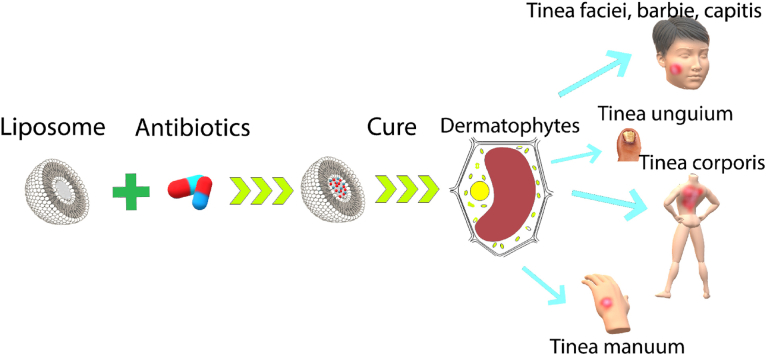

## 1 Introduction

Fungi are naturally present on the skin of the human body. These fungi do not cause any particular problems to the health of the body and skin. However, under certain conditions, these harmless fungi can begin to grow and multiply irregularly [[1], [2], [3]]. There are also cases where pathogenic fungi penetrate the skin and cause fungal infections. Dermatophytes are a group of fungi that are particularly interested in keratin and therefore attack the skin, hair, and nails, leading to fungal infections [4]. Every year, millions of people suffer from dermatophytosis, an infection caused by dermatophyte fungi [5,6]. Approximately 30%–70% of adults carry dermatophytes without clinical symptoms, and at least 10% of people develop dermatophytosis during their lifetimes [7,8]. For the treatment of fungal infections, the use of topical and oral antifungal drugs can be useful [9]. However, there are disadvantages to using chemical medications. These disadvantages include drug wastage, unwanted side effects (such as diarrhea and upset stomach), high raw material costs, physicochemical incompatibilities, and clinical drug interactions [[10], [11], [12]]. A major challenge is also that the frequent use of conventional medications and antibiotics leads to the emergence of antibiotic-resistant microorganisms and the formation of biofilms [3,13,14]. Recent advances in medical microbiology have led to the development of new diagnostic tools and treatments, which have the potential to revolutionize the field of infectious disease management [[15], [16], [17], [18], [19]]. Recently, researchers have developed nano-formulations based on nanomedicine to effectively treat dermatophytosis and reduce the adverse effects of chemical therapeutic agents [[20], [21], [22]].

Nanotechnology gained lots of interest in recent years in the field of diagnosis and treatment of different disorders [23]. Also, there are many reports of the use of nanotechnology in the field of microbiology and antibiotic resistance [24,25]. Using nanotechnology, a targeted drug delivery system can be utilized to specifically target the affected area. Additionally, the nano-drug delivery system generally involves the controlled and precise delivery of the drug to the targeted tissue with the assistance of nanocarriers. The drugs can be embedded in the structure or on the surface of the nanocarriers and released into the target tissue at appropriate therapeutic concentrations [26,27]. Furthermore, it serves to enhance the efficacy of drugs, diminish their adverse effects and toxicity, and enhance their stability. This field can be utilized for various predefined purposes. Nanotechnology is an exciting scientific field where new discoveries are made every day around the world, and its potential is limitless [28,29]. In recent years, scientists have utilized nanotechnology to develop a new approach in making drugs smarter, thereby enhancing their effectiveness in achieving the desired goals [[30], [31], [32], [33]]. Nanoparticles can be based on metallic nanoparticles, phospholipid vesicles (liposomes, autosomes, and transfersomes), non-phospholipid vesicles (niosomes), solid lipid nanoparticles (SLNs), and other nanostructures. Among these, lipid-based nanosystems, including liposomes, SLNs, and nanostructured lipid carriers, are the most suitable systems for antifungal delivery [[34], [35], [36], [37]].

Liposomes are self-forming nanostructures that result from the arrangement of lipid molecules in an aqueous solution. The use of liposomes for oral drug administration, as well as for application on the skin, eyes, and blood vessels, has been extensively studied. The liposomes developed for these purposes can either deliver the drug specifically to the target tissue, known as targeted liposomes, or act solely as carriers and reservoirs. Liposomes coated with active polymers exhibit advantageous properties, such as improved mucosal adhesion and enhanced permeability of the gastrointestinal wall [[38], [39], [40], [41], [42], [43]]. Today, these nanostructures are used as carriers for drugs, antibiotics, and genes, as well as for modeling cell membranes in animals and humans. Additionally, liposomes interact with the skin and can be absorbed in various ways. They can be absorbed onto the skin's surface, releasing the drug in the process. Moreover, they can penetrate the epidermal layer through lipid channels to treat superficial infections. The penetration of the drug delivery system through the skin is crucial for effectively treating superficial infections, as the drug needs to reach the stratum corneum where the fungi reside. However, the skin's inhibitory properties often hinder drug penetration. Recently, the topical delivery of antifungals using lipid vesicles has demonstrated significant potential [[44], [45], [46], [47], [48]].

Several antifungal compounds were formulated as liposomes, and their characteristic properties, efficacy, and suitability were evaluated. The results of one study showed that liposomes loaded with amphotericin B displayed a lower minimum inhibitory concentration (MIC) compared to separate applications of amphotericin B and liposomes [49]. In another study, the authors evaluated the antifungal susceptibility of liposomal fluconazole compared to conventional fluconazole on dermatophyte isolates. According to the data, the MIC50 for the three examined species, *Trichophyton interdigitale*, *T. rubrum*, and *Epidermophyton floccosum*, was 32, 16, and 8 μg/mL, respectively. The corresponding values for nano fluconazole were 8 μg/mL for all three tested species [50].

Terbinafine-loaded liposome formulations were developed by Sudhakar et al. (2014). The results showed that these formulations act as effective antifungal agents containing terbinafine hydrochloride [51].

**Fig. 1:**
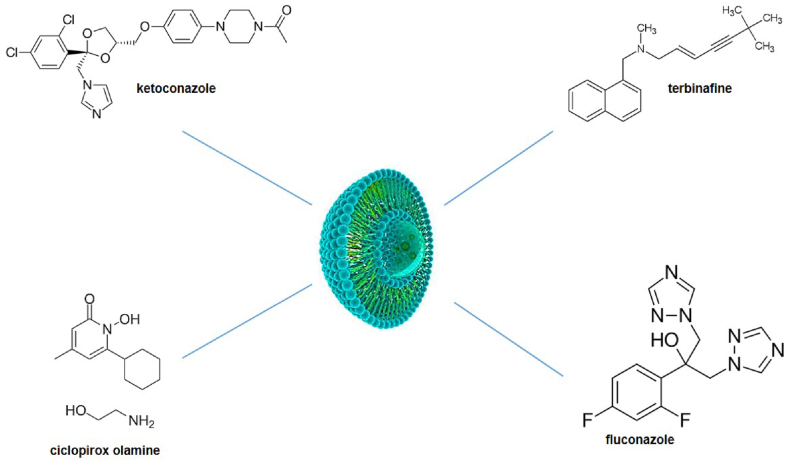
Liposomal nanoformulation with some important and widely used antifungal topical drugs.

Finally, newer formulations of liposomes have recently been developed. Fig. 1 depicts the liposomal nanoformulation with several important and widely used antifungal topical drugs. The objective of this review article is to assess the recent advancements in liposomal formulations for the treatment of dermatophytes. These formulations exhibit greater efficacy, resulting in increased efficiency.

## 2 Dermatophytes and characteristics

### 2.1 Classification

Dermatophytes are the primary causative agents of dermatophytosis. One of their most important characteristics is their ability to thrive on keratin. The three closely related genera of dermatophytes are Epidermophyton, Trichophyton, and Microsporum. Dermatophytes can be classified into three ecological groups based on their sources and habitats. They can originate from human skin (anthropophilic), animals (zoophilic), or soil (geophilic). Geophilic dermatophytes are typically associated with decomposed keratinous materials, such as wool, hair, feathers, pelts, hooves, nails, horns, cartilage, etc., after they have been separated from living animals. They can infect both humans and animals. Zoophilic pathogens primarily affect animals but can occasionally infect humans as well. Anthropophilic organisms predominantly infect humans and rarely affect animals. Since dermatophytosis is contagious, direct human contact is usually required for transmission. Despite the geophilic origins of anthropophilic and zoophilic dermatophytes, the anthropophilic group is highly specialized. The most prevalent dermatophytes worldwide associated with human dermatophytosis include *T. mentagrophytes*, *T. rubrum*, *T. tonsurans*, *M. canis*, and *E. floccosum*. Some less common dermatophytes that can cause mycosis in humans include *T. verrucosum*, *M. gypseum*, and *T. soudanense* [52,53].

### 2.2 Pathogenesis

Dermatophytes primarily inhabit the dead keratinized tissue of the stratum corneum, as well as hair and nails. The growth of dermatophytes in these tissues leads to the formation of hyphae and arthroconidia, which serve as diagnostic features. When dermatophytes invade the epidermis, they follow a common pattern of invasion. It starts with the adhesion of arthroconidia to keratinocytes, followed by penetration through and between cells, ultimately triggering a host response [54,55]. Dermatophyte infections can spread through direct contact with an infected individual or animal, as well as through the use of contaminated objects such as clothing and furniture. Several factors contribute to the pathogenicity of dermatophytes, including the production of keratinolytic enzymes, host factors, and genetic predisposition. Dermatophytosis has been associated with various host factors, including diabetes mellitus, circulatory disorders, psoriasis, ichthyosis, immune system disorders, HIV infection, and more. Skin-related factors play a crucial role in the development of clinical dermatophytosis [55,56]. The size and duration of the lesion in the stratum corneum depend on the growth rate of the organism and the turnover of the epidermis. Infections of the hyponychium and distal nail bed in the stratum corneum typically lead to onychomycosis, which then spreads proximally into the nail bed and affects the ventral surface of the nail plate. Dermatophytes can enter nails through two pathways: 1) by entering the eponychium and growing within the soft keratin layer on the ventral side of the nail, and 2) by entering the ventral nail groove, crossing the nail plate, and ultimately reaching the ventral layer. Dermatophytes invade the hair follicle from the adjacent stratum corneum. They descend within the inner part of the hair until they reach the boundary of the keratogenous zone. At this location, a tuft of hyphae known as Adamson's fringe is present [54,55,57].

### 2.3 Clinical manifestations

In dermatophyte infections, the combination of keratin disruption and inflammatory host responses gives rise to clinical features. Dermatophytosis commonly presents as an inflamed scaling patch with a raised margin that exhibits varying degrees of inflammation, with the center of the patch typically being less inflamed than its margin. The clinical presentation can vary significantly depending on the species and strain of the fungus, the site of infection, the size of the inoculum, and the immune status of the host. There are eight types of dermatophytosis commonly observed in clinical settings: tinea capitis (involving the hair/scalp), tinea corporis (involving glabrous skin), tinea cruris (involving the groin), tinea faciei (involving the face), tinea manuum (involving the hands), tinea pedis (involving the feet), tinea barbae (involving the beard area), and tinea unguium or onychomycosis (involving the nails). Besides fungi being a significant factor in the transmission of tinea, host characteristics such as local conditions and immunological status, including excessive moisture, trauma, or occlusive clothing, may contribute to the risk of infection when combined with exposure to fungi [56,57].

**Fig. 2:**
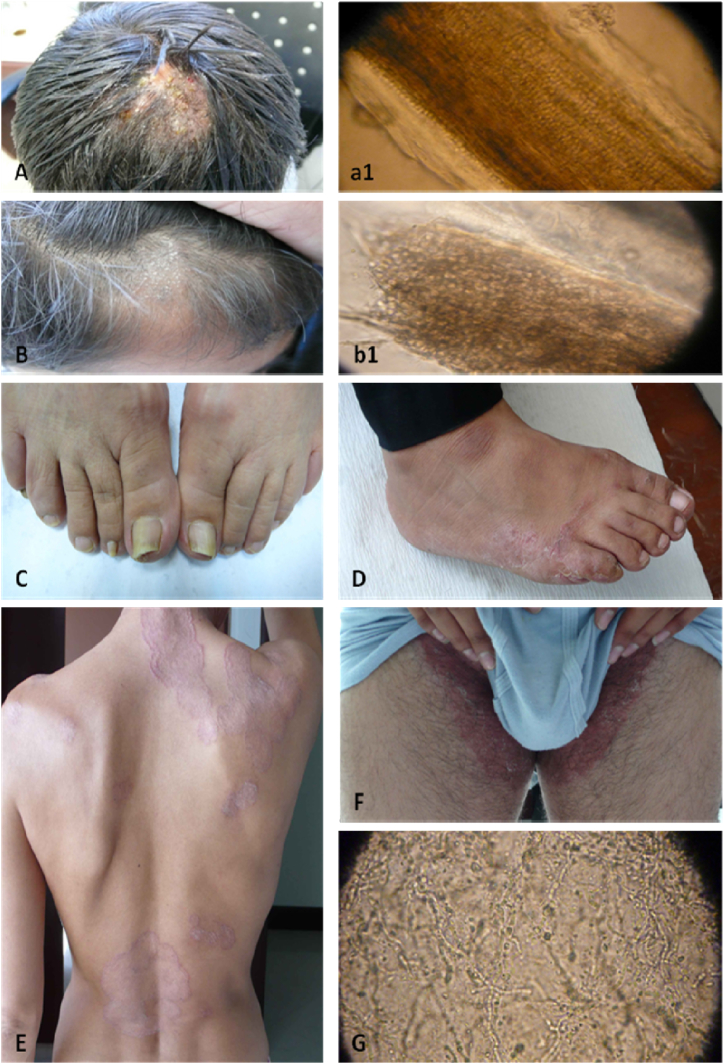
Clinical manifestations of dermatophytosis. (A) Clinical aspects of tinea capitis (Endothrix), (a1) microscopic aspect of Endothrix, (B) Clinical aspects of tinea capitis (Ectothrix), (b1) microscopic aspect of Ectothrix, (C) Clinical aspects of tinea unguium, (D) Clinical aspects of tinea pedis (E) Clinical aspects of tinea corporis (F) Clinical aspects of tinea cruris (G) microscopic aspect of dermatophyte.

**Tinea capitis (ringworm of the scalp):** Dermatophytes primarily affect the scalp, hair, and hair follicles. Trichophyton and Microsporum are the main causative agents of this disease. Depending on the species infecting the hair, spores can either form within the hair shaft or on its surface. Endothrix infection occurs when spores form within the hair, leading to significant weakening of the hair shaft (Fig. 1, Fig. 2). Endothrix infections are most commonly caused by *T. tonsurans* and *T. violaceum*. On the other hand, ectothrix infection is caused by dermatophytes that invade the hair shaft externally and destroy the cuticle (Fig. 2, B & b1). Symptoms of dermatophyte infections of the scalp include fine scaling with circular alopecia (gray patches) and the presence of single or multiple scaly plaques.

There are four distinct clinical patterns of tinea capitis: the black dot pattern, kerion pattern, favus pattern, and seborrheic pattern. Kerion and favus are inflammatory forms of tinea capitis. Kerion is typically caused by zoophilic dermatophytes such as *T. verrucosum* or *T. mentagrophytes*. Favus, also known as tinea favosa, initially presents with perifollicular erythema and subsequently develops honey-colored concave or cup-shaped follicular crusts (scutula) that are clustered like honeycombs on the scalp. *T. schoenleinii* is the most commonly isolated dermatophyte associated with favus [52,53,58].

**Tinea corporis (Tinea circinata, Tinea labrosa, Ringworm of the body):** Tinea corporis affects the shoulders, trunk, limbs, and occasionally the face (excluding the area around the beard) (Fig. 2, E). The three most common causative organisms are *T. rubrum*, *M. canis*, and *T. mentagrophytes*, although regional variations may exist due to endemic species. The clinical appearance of tinea corporis varies depending on the causative organism. The characteristic lesion is a circular red or pink patch with a raised, well-defined border. In cases of inflammatory forms, which are often caused by zoophilic or geophilic dermatophytes, papules, crusts, or even pustules may develop along the advancing border. The anthropophilic dermatophyte *T. rubrum* can cause chronic, non-inflammatory, extensive lesions. The level of inflammation is influenced by various factors, including the type of fungus involved, the host's immune system, and the extent of follicular invasion [52,57].

**Tinea cruris (Jock itch, Dhobi itch, Ringworm of the groin):** Tinea cruris is caused by dermatophytes that affect the inguinal region of the body (Fig. 2, F). The skin of the genitalia, pubic area, perineal area, and occasionally the upper thighs can be affected. Depending on the severity, tinea cruris can range from acute to chronic inflammation of the skin. The most common causative agent of tinea cruris is *T. rubrum*, while *E. floccosum* and *T. mentagrophytes* are also isolated in dermatophyte infections of the groin. This infection is highly contagious and is typically transmitted through contact with contaminated towels or by touching contaminated surfaces in hotel rooms, bathrooms, or showers. Many individuals with tinea cruris also develop concomitant tinea pedis, caused by the same fungi. Children are less likely to be infected compared to adults, and it is three times more common in men than in women. However, tinea cruris has become more prevalent among overweight women who wear tight-fitting clothing [52,53,59].

**Tinea Pedis (Foot Ringworm, Athlete's foot):** An increasing number of people are experiencing tinea pedis, commonly known as athlete's foot. This condition is often caused by anthropophilic species such as *T. rubrum*, *T. interdigitale*, and *E. floccosum*. Infections caused by *T. rubrum* typically result in chronic non-inflammatory erythematosquamous lesions, while infections caused by *T. interdigitale* often lead to vesicular or bullous inflammatory lesions. Tinea pedis commonly affects both feet (bilaterally) rather than just one foot (unilaterally), and it is most prevalent in men aged between 20 and 50. In societies where shoes are not frequently worn, the prevalence of the infection may be lower. Exposure to a moist environment and skin maceration are significant predisposing factors for acquiring tinea pedis. The clinical manifestations of tinea pedis include four main forms: interdigital, erythematous, vesicular, and ulcerative. The most common clinical form is interdigital tinea pedis (athlete's foot), which primarily affects the spaces between the toes (Fig. 2, D) [52,56,57].

**Tinea manuum (Ringworm of the hand):** Tinea manuum is an infection that affects the skin of the hands and the interdigital skin. The most common cause of this infection is *T. rubrum*. Ringworm of the glabrous skin refers to an infection on the dorsal (top) surface of the hand. A dorsal lesion exhibits similarities to Tinea corporis. Non-inflammatory lesions are characterized by mild scaling or diffuse scaling hyperkeratosis, often accompanied by fissures in the palmar crease. Inflammatory lesions are typically multiloculated and primarily develop on the palms, rarely on the dorsum [57,59].

**Tinea unguium (Ringworm of the nails, Onychomycosis):** In tinea unguium, the nail plate becomes infected by dermatophytes (Fig. 2, C and G). The term onychomycosis is commonly used to describe nail infections. The first and fifth toenails are most frequently affected, although other nails can also be involved. Toenail onychomycosis is more prevalent than fingernail onychomycosis and often results from tinea pedis (athlete's foot), while infected fingernails can associate with tinea manuum, tinea capitis, and tinea corporis. The most common causative agents for this nail disease are *T. mentagrophytes* and *T. rubrum*. Additionally, *E. floccosum*, *T. tonsurans*, and *T. verrucosum* may be responsible, although the latter is typically found in fingernails. Onychomycosis can occur at any age, but it is more common in older individuals, affecting both males and females equally. Generally, there are three types of onychomycosis: distal subungual, white superficial and proximal subungual. Distal subungual onychomycosis is the most common form and primarily affects the nail beds rather than the nail plate.

**Tinea faciei (Ringworm of the face, “Tinea corporis of the glabrous skin of the face”):** Infections caused by dermatophytes on the glabrous skin of the face are relatively common. *T. rubrum* and *T. mentagrophytes* are frequent causative agents. The specific causative agent may vary depending on the geographic region, but typically the infection originates from a zoophilic reservoir in animals and pets, or it can be an extension of the infection from another part of the body [59].

**Tinea barbae (Tinea sycosis, Barber's itch, Ringworm of the beard):** A ringworm infestation can occur in the beard and mustache area of the face, leading to an invasion of coarse hairs. The lesions can present with various characteristics, ranging from inflammatory pustules to non-inflammatory scaly patches. The infection is typically caused by two zoophilic fungi, *T. verrucosum* and *T. mentagrophytes*, while *M. canis* is less commonly involved [59,60].

### 2.4 Epidemiology

A high prevalence rate of dermatophytic infections has revealed that 20–25% of the world's population suffers from skin mycosis. Dermatomycosis is the most common form of mycosis in most parts of the world. The prevalence of dermatophytosis can vary significantly based on the climatic conditions and lifestyle of a particular location. The development of dermatophytosis is supported by tropical and subtropical environments. While dermatophytosis is prevalent globally, certain varieties are found only in specific areas. For example, *M. ferrugineum* is largely restricted to Asia and Africa, *T. soudanense* is endemic to Africa, *T. yaoundei* is found in central parts of Africa, and *T. concentricum* is mainly restricted to the western Pacific Islands and Southeast Asia [52,53].

## 3 Current diagnosis options

Distinguishing dermatophytosis from other non-mycotic dermatoses can be challenging, especially in cases of dystrophic nails. Therefore, establishing an accurate biological diagnosis is crucial. Before initiating antifungal therapy, it is important to confirm that the infection is indeed caused by dermatophytes. The conventional method for identifying dermatophytes involves macroscopic and microscopic examination of hyphae/spores from lesional material as well as in vitro cultures [52].

### 3.1 Sampling

For an accurate diagnosis of fungal pathogens, it is essential to obtain an adequate sample. However, sample collection techniques and transportation devices used for transport can potentially contain environmental fungi that may cause contamination. Therefore, it is recommended to clean the lesion area with a 70% alcoholic solution before collecting the sample to remove contaminants.

The sites from which samples are collected depend on the clinical symptoms presented by the patient. When ringworm lesions are suspected, skin scrapings are taken from multiple areas of the patient's body. In cases of onychomycosis, fingernail and toenail clippings are collected. For tinea capitis and tinea barbae, a hair sample and scrapings from the lesions are obtained [61,62].

### 3.2 Direct microscopy examination

In direct microscopy, slides are prepared by treating skin samples with 20% KOH, nail clippings with KOH + DMSO, and hairs with lactophenol. Dermatophytes are characterized by hyaline, septate, branched, and unbranched hyphae that have arthroconidia. Direct examination can sometimes be challenging to visualize the fungal elements, but staining techniques can aid in the visualization of the fungal structures. Various stains, along with clearing agents, can be used for direct examination. For example, stains such as Calcofluor, chlorazol black, cotton blue C4B, Congo red, and blue-black ink can be employed to assist in the detection of fungal elements [[61], [62], [63]].

### 3.3 Culture

In the diagnosis of dermatophyte infections, a fungal culture is necessary. Clinical materials such as skin scrapings nail clippings, and hair samples are cultured on mycological culture media, either in slant tubes or on plates. The most commonly used culture media for dermatophyte isolation are mycobiotic agar, dermatophyte test agar, and Sabouraud agar with Chloramphenicol and Cycloheximide (SCC). The inoculated cultures are incubated aerobically at 25–30 °C for four weeks and checked weekly. Identification of the grown dermatophytes is based on macroscopic and microscopic characteristics, hair perforation tests, urease tests, and cornmeal agar tests [61,62].

### 3.4 Wood light examination

Wood's lamps are handheld lamps that emit ultraviolet radiation through a nickel or cobalt glass filter. They can be used as a screening tool for diagnosing dermatophytosis, although they are not very helpful and a positive or negative result does not definitively rule out an infection. The main purpose of the procedure is to aid in identifying hairs for culture or direct examination. When ultraviolet light is projected through a Wood's filter, it produces light rays with a wavelength above 365 nm. If the hair is infected with *M. canis* or *M. audouinii*, it will fluoresce blue-green under the Wood's lamp, while the scalp will not fluoresce. Infected hair caused by the rare dermatophyte *T. schoenleinii* may exhibit a mild green fluorescence. No other dermatophytes that infect the hair produce fluorescence. It is important to note that fungal infections of the skin do not produce any fluorescence under Wood's lamp examination [61,64].

### 3.5 Molecular detection and identification

Molecular biology techniques have significantly advanced the detection and identification of dermatophytes in the investigation of superficial mycosis in recent years. To identify dermatophytes using molecular techniques, DNA is extracted directly from infected tissue or obtained from isolated cultures. These molecular tools enable the diagnosis of dermatophytosis within 48 h. There are three types of molecular diagnostics: conventional PCR, real-time PCR, and methods utilizing post-PCR techniques.

Conventional PCR is relatively inexpensive and straightforward to perform, making it a popular choice. Real-time PCR assays are highly desirable as they allow for automated analysis in a closed tubing system, reducing the risk of contamination and enabling simultaneous detection of multiple species. Since 2010, studies have examined the utility of various post-PCR techniques for the detection of dermatophytes in clinical specimens, all of which have highlighted the benefits of their high sensitivities [65,66].

Nested PCR is a technique in which a conventional PCR is followed by a subsequent amplification of a smaller region within the initially amplified fragment of biological material. In the case of dermatophyte detection, the primers used in this method target a pan-dermatophyte-specific sequence. According to Garg et al. the pan-dermatophyte nested PCR has been proposed as a potential gold standard for diagnosing dermatomycosis in nails due to its higher sensitivity compared to KOH microscopy [67,68].

Multiplex real-time PCR is a method developed by Arabatzis et al. for the detection of dermatophytes in clinical specimens of nails and skin. This technique utilizes the ITS1 and ITS2 regions as targets for amplification. Researchers have successfully developed qPCR assays that allow for rapid detection and identification of clinically relevant species of dermatophyte genera in samples from nails, skin, and hair [69]. A multiplex PCR assay utilizing chitin synthase I and the ITS region was developed in 2014 for the detection and identification of *T. rubrum* and *T. mentagrophytes* in nail samples. This method exhibited a sensitivity of 97%, which is higher compared to conventional methods [70].

The PCR reverse-line blot assay (PCR-RLB) was developed in 2008 and is based on ITS sequences. This test allows for the detection and identification of dermatophyte species in skin, hair, and nail samples within a few hours. The method has demonstrated good sensitivity and specificity. However, it should be noted that diagnostic settings can be high-risk and labor-intensive, and amplicon contamination is a common concern [53].

The 24-h PCR ELISA method involves amplifying the topoisomerase II gene region using PCR and then hybridizing it to arrays of biotin-labeled probes using digoxigenin-labeled PCR products. When compared to fungal cultures, this method demonstrates a sensitivity of 90% and exhibits good specificity, as no cross-hybridization was observed with any dermatophyte species or human DNA [53,71].

PCR-RFLP (Restriction Fragment Length Polymorphism) is a method that allows for the direct detection of dermatophytes in skin, hair, or nail samples. The method relies on the use of various restriction enzymes that generate distinct fragment patterns upon digestion, which can differentiate between different species or strains. With this method, successful detection of *T. mentagrophytes*, *T. rubrum*, and *E. floccosum* has been achieved; however, strain differentiation was not observed among them [72].

## 4 Current treatment options

The type and duration of treatment vary depending on the infecting organism, the site of infection, the severity of the illness, and other patient-specific factors such as concurrent illnesses and medications [57]. An overview of the most common antifungal agents used to treat dermatophytes and their adverse effects is provided in Table 1.

**Table 1:** Conventional antifungal agents for Dermatophyte Infections and adverse effects.

Antifungal agents	Central nervous system	Gastrointestinal	Dermatological	Liver	Blood disorders	Other	Reference
**Griseofulvin**	•headachee •Lethargy •Irritability •Nightmares	•Nausea •Vomiting •Diarrhea •Dryness of the mouth •Taste disturbances	•Urticaria •Erythema multiforme •Necrolysis •Exanthems •Dermatitis •Exacerbation or unmasking of lupus erythematosus	•liver function abnormalities	•Leukopenia	•carcinogenic and teratogenic in animal models	[73]
**Terbinafine**	•Headache	•Nausea •Diarrhea	•Rash •Pruritus •Eczema •Stevens-johnson syndrome •Necrolysis •Cutaneous or systemic lupus erythematosus	•liver failure	–	•Visual disturbances •Dysgeusia •Mild transaminitis	[74]
**Itraconazole**	•Headache	•Nausea •Vomiting •Diarrhea •Flatulence •Anorexia •Dry mouth	•Rash •Pruritus,	•liver function abnormalities	•Thrombocytopenia	•Hypertriglyceridemia •Hypokalemia •Oedema •Polyuria •Decreased libido •Gynaecomastia •Impotence	[75]
**Fluconazole**	•Headache	•omitting •Diarrhea •Abdominal pain •Nausea •Taste disturbances	•Rash	–	•Purpura	–	[76,77]

### 4.1 Treatment of tinea capitis and tinea barbae

While the reviewed papers primarily focus on the simultaneous determination of multiple azole drugs in biological samples, it is worth considering recent quantitative procedures applied to antifungal topical drugs in general. Such procedures can offer valuable insights into the utilization and effectiveness of these drugs, especially in the treatment of dermatophyte infections. Several recent studies have explored the quantification of antifungal topical drugs in biological fluids and complex matrices [78,79].

Systemic antifungal therapy is utilized for the treatment of tinea capitis and tinea barbae. The preferred drug of choice is griseofulvin, typically administered at a dose of 15–25 mg/kg per day. Griseofulvin is best taken with a fatty meal or milk to enhance absorption. Treatment usually continues until clinical and mycological cure is achieved, which may take up to 8 weeks in some cases.

Other effective agents for these conditions include terbinafine (250 mg/day for individuals weighing over 40 kg, 125 mg/day for those weighing 20–40 kg, and 62.5 mg/day for individuals weighing less than 20 kg), itraconazole (3–5 mg/kg/day), and fluconazole (3–6 mg/kg/day for 6 weeks). There have also been reports of terbinafine and itraconazole pulse therapy.

Patients with inflammatory tinea capitis may benefit from the addition of systemic or topical corticosteroids to prevent scarring and permanent hair loss [57,58,80].

### 4.2 Treatment of tinea corporis tinea cruris, tinea faciei and tinea pedis

These types of infections are typically treated with topical agents. The agents should be applied twice daily for 2–4 weeks. According to reports, allylamine terbinafine has shown effectiveness within 1 week of therapy, possibly due to its fungicidal properties. Patients with extensive disease, inflammatory infections, or compromised immune systems may benefit from adjunctive treatment with ketoconazole, griseofulvin, or one of the triazoles (fluconazole or itraconazole). The use of terbinafine as adjunctive therapy may also be beneficial. Additionally, a topical corticosteroid can provide relief from inflammation and itching [60].

### 4.3 Treatment of tinea unguium

Tinea unguium (nail fungus) is rarely responsive to topical therapy, and spontaneous healing is uncommon. The preferred treatment for tinea unguium is oral terbinafine. Terbinafine is administered at a dose of 250 mg/day for six weeks for fingernail infections and 12 weeks for toenail infections. Additionally, it is recommended to apply tioconazole or amorolfine topically for at least 6 months for fingernail infections and 12 months for toenail infections. Another option is itraconazole (200 mg/day) taken for 12 weeks for toenail infections, with or without fingernail involvement [57].

## 5 Liposome as a new option for dermatophyte infections

**Fig. 3:**
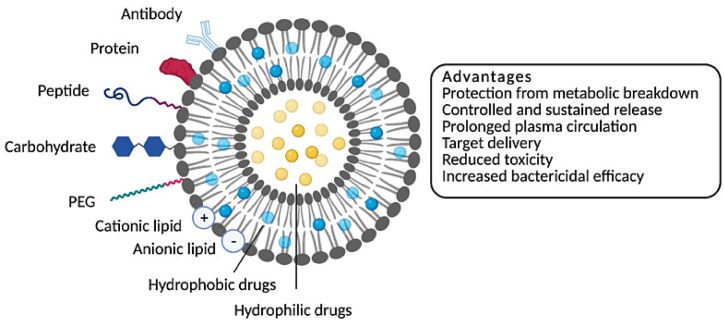
Different forms of liposomes are shown in a schematic diagram, along with their different targeting approaches [88].

There are many new advances in the field of nanotechnology for the treatment of microbial infections [[81], [82], [83], [84]]. By the way, lipid-based nano-systems, such as liposomes, have demonstrated particularly appealing physicochemical properties and safety profiles compared to other nanocarriers. Liposomes are spherical structures composed of one or more phospholipid bilayers encapsulating aqueous compartments [42,85]. Fig. 3 presents a schematic illustration of various liposome forms and their key advantages. The ability of liposomes to undergo surface modification is a crucial attribute that positions them as potential groundbreaking treatments for antifungal applications, as further discussed below. Surface functionalization using ligands, such as molecules or polymers, enables targeted delivery of antibiotics, which is crucial for achieving effective drug delivery and therapeutic outcomes [86,87].

**Fig. 4:**
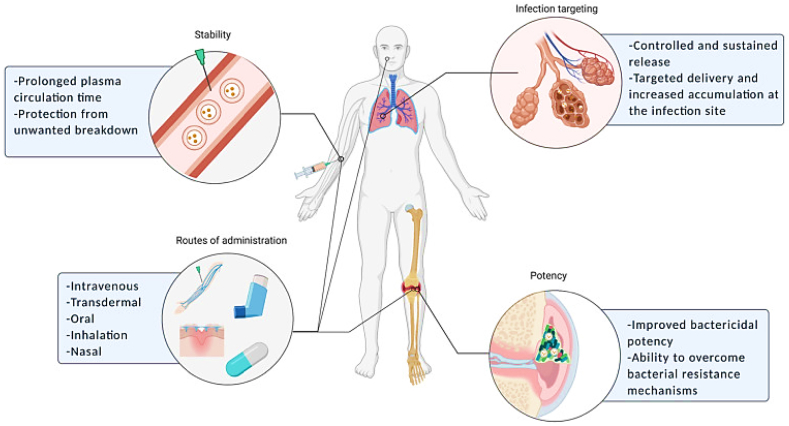
Liposomes as antifungal carriers: a graphic illustration of the primary benefits of liposomes [88].

The advancement of enhanced liposomal formulations has opened up possibilities for the development of potential antifungal delivery systems that can address critical challenges in the management of pathogenic fungi. The utilization of liposomes as nanostructures for delivering antifungal agents offers several advantages, including the capability to overcome issues related to drug effectiveness and the emergence of resistant organisms. Fig. 4 provides a graphical representation highlighting the key benefits of liposomes as carriers for antifungal therapies.

### 5.1 Liposome as an effective tool for the management of dermatophytes infections

Topical ointments and systemic administration are both viable options for treating dermatophyte infections in the general population. Examples of topical medications include clotrimazole, butenafine, miconazole, and terbinafine. For systemic treatment, oral medications such as fluconazole, griseofulvin, terbinafine, and itraconazole are commonly used. Both itraconazole and terbinafine have been identified as potential agents for expediting the healing process of severe skin lesions. However, terbinafine is generally favored over itraconazole due to its comparatively lower occurrence of adverse effects [89,90]. One of the major challenges in standard therapies for dermatophyte disease is the poor absorption rate and the requirement for prolonged administration of these medications [89]. Several liposomal formulations have been developed to enhance the effectiveness of standard therapies in the context of dermatophyte infections. The use of liposomal formulations for topical and oral administration of antifungal drugs in the treatment of dermatophyte infections will be discussed in further detail in the following paragraphs.

#### 5.1.1 Topical medications

The administration of drugs through the skin appears to be the most effective method for treating dermatophyte infections. Topical formulations can release a significant amount of drugs in therapeutic concentrations, allowing the drug to penetrate deeper layers of the skin, including the stratum corneum. The stratum corneum, which is the outermost layer of the epidermis, acts as a barrier to prevent drug permeation into the skin [89]. A variety of functions are demonstrated by liposomes, especially when they are nanosized and used as topical drug carriers for the skin. These functions depend on the size of the liposomes, their lamellarity, the percentage of ingredients present, their lipid and cholesterol composition, and their surface charge [91]. By localizing their activities, liposomes have the potential to reduce systemic effects, enhance penetration through the entire stratum corneum, and overcome various challenges in cutaneous drug delivery. Encapsulating antifungal agents in liposomes can effectively promote skin permeation, localize drug activity, and improve drug delivery performance [92]. For example, griseofulvin is an antifungal medicine that is available in oral and injectable dose forms [93]. The development of topical treatments for superficial fungal skin infections holds potential benefits. A study examined the use of films created by incorporating liposomes loaded with griseofulvin into chitosan film for topical administration in such infections. The study focused on assessing important characteristics of the films for their antifungal efficacy against *E. floccosum* and *M. gypseum*. These characteristics included swelling, vapor transmission ability, mechanical properties, thermal behaviors, drug release, and antifungal effectiveness. The presence of liposomes in the films resulted in diminished mechanical properties but reduced swelling ratio. Comparing liposomal formulations to liposomal films, it was observed that liposomes incorporated in films exhibited higher levels of drug permeation and a faster flux rate. The antifungal effectiveness of the formulations was demonstrated in vitro against the two species of dermatophytes, as previously reported. Ultimately, the authors combined the vesicular delivery system with the biopolymeric film, resulting in a topical composite film that showed excellent potential for delivering griseofulvin to superficial fungal infections [94].

In addition to being a fungal infection of the nail unit, onychomycosis is caused by dermatophytes. Terbinafine hydrochloride (TBF-HCl), an oral medication, has been used for over twenty-four years to treat onychomycosis [95]. The motivation behind the creation of a new formulation utilizing TBF-HCl-loaded liposomes stemmed from the adverse effects linked to the systemic administration of this medication. To describe the newly prepared film formulations, various characteristics were measured, including thickness, bioadhesive capabilities, physical appearance, drug content, and tensile strength. A series of in vitro and ex vivo permeation tests were conducted to determine the most effective film formulation with antimicrobial properties and to demonstrate the efficacy of different formulations in treating onychomycosis. In vitro, the liposome-loaded pullulan films (LI-P, LII-P) and liposome-loaded Eudragit films (LI-E, LII-E) exhibited drug release percentages of 71.6% and 54.4%, respectively. The accumulated drug in the nail plates for LI-P, LII-P, LI-E, and LII-E was found to be 31.16, 24.81, 8.17, and 8.92, respectively, all within the therapeutic range for all film formulations. Comparisons were made between TBF-HCl-loaded liposome film formulations and ethosome, liposome poloxamer gel, TBF-HCl-loaded liposome, and ethosome chitosan gel formulations to assess performance. TBF-HCl-loaded liposome film formulations were found to exhibit superior antifungal activity on fungal nails, making them an attractive option for the treatment of fungal nail infections [96].

In general, nanocolloids enhance drug absorption through the skin. However, the skin contact and primary drug diffusion channels may vary depending on the platform and formulation characteristics. Understanding the interaction between different colloidal structures and biomembranes, as well as the impact of formulation on distribution, is particularly crucial for drugs such as voriconazole, which can be encapsulated in various nanosystems [44]. In this context, the release and permeation profiles of voriconazole were examined in gel formulations and aqueous colloidal systems, specifically nanostructured lipid carriers (NLC) and liposomes (LP). While gel formulations provided controlled drug release, they had minimal influence on LP's ability to accumulate in the skin. However, drug retention and follicle deposition in the skin were affected by the limited mobility of gel formulations. Permeation investigations, particularly through the follicular route, supported this observation. The follicular route had an impact on NLC delivery, mainly in terms of the overall amount of medication reaching the acceptor medium. These differences may be attributed to the processes of colloid-skin contact and subsequent drug release. LP deposition in follicles and delayed drug release led to higher levels of drug in the skin, while NLC contact with the skin and rapid drug release facilitated faster diffusion and deeper penetration. Regardless of the system, the permeated quantities of VOR exhibited potential for limiting fungal growth, as evidenced by the low MIC50 values observed against *T. rubrum* (0.001 g/mL). In conclusion, both LP and NLC appear to be promising strategies for delivering voriconazole to the skin, with fluidic formulations from LP showing greater efficiency in cutaneous medication administration [44].

Another study focused on designing a formulation of itraconazole loaded liposomes (LPs) for effective topical application against dermatophyte infections. A two-step design of experiments (DoE) strategy was employed to optimize the formulation. This involved a fractional factorial design to test key variables and surface response mapping, which was further optimized using fractional factorial and full factorial analysis methods. The goal was to achieve the optimal itraconazole-loaded liposome (OPT-NLPs) suspension by considering vesicle size, itraconazole skin retention, percent drug entrapment (PDE), and permeation through response maps. To improve topical applicability, the liposome suspension was transformed into a gel form. The OPT-NLPs formulation exhibited high PDE (78.69 ± 3.17%), with a mean zeta potential of 20.66 mV and an average vesicle size of 358.2 nm. Furthermore, in comparison to an oily itraconazole solution and conventional cream, excised rat skin demonstrated significantly higher itraconazole skin retention (44.39 g/cm^2^) and cumulative 6-h drug permeation (14.81 g/cm^2^). Additionally, a standardized tinea pedis animal model was utilized to evaluate the in vivo antifungal effectiveness of the formulation, assessed by fungal burden score, physical manifestations, and histopathological profiles. Animals treated with the optimized hydrogel exhibited faster relief from the infection compared to those treated with commercial topical and oral antifungal treatments. Based on the findings, the developed formulation holds promise as a potential and expedient alternative to traditional antifungal treatments for dermatophytosis [97].

A comparison was made between a topical formulation of 1% liposome TH cream and a standard TH formulation. A synthetic membrane was employed to assess permeation. The study investigated the penetration dynamics within the layers of the stratum corneum for both formulations, as well as the percentage of drug release and diffusion rate. The results showed that the liposome TH cream exhibited a higher ability to release the active component throughout a larger proportion of the stratum corneum layers, providing a greater dose of the drug. This finding is highly encouraging for the treatment of superficial mycoses [98].

Luliconazole is a promising new drug for the treatment of skin fungal infections. However, its current therapy is limited by slow skin absorption, requiring long-term use and repeated doses for effective treatment due to its poor solubility. To address these limitations, elastic ethogel and lipo gel formulations based on lipid nanosystems were developed and extensively studied in vitro, ex vivo, and in vivo, with comparison to a commercial formulation. The produced formulations exhibited nanometric vesicle size, optimal pH, zeta potential of 17.0 and 32.8 mV, high encapsulation efficiency of 92.7% and 91.2%, and suitable viscosity (6.6 and 7.8 Pa s). Stability was maintained during storage without any signs of instability. In vitro studies demonstrated that the developed formulations were 2.5 and 3 times more effective than the commercial formulations against dermatophytes and *Candida albicans*, respectively. Ex vivo skin deposition and permeation tests on synthetic and biological membranes revealed that the Strat-M membrane closely resembled human skin. Scanning electron microscopy (SEM) and confocal laser scanning microscopy (CLSM) analysis confirmed enhanced skin deposition of the elastic ethogel and lipo gel compared to the ordinary marketed cream. In vivo antimicrobial evaluation in albino rats demonstrated that the vesicle-based gel formulations were safe, non-irritating, and more efficient in the topical treatment of fungal infections without the drug entering the bloodstream. Based on the findings of the study, elastic ethosomes and liposomes as a vehicle offer an attractive option for improving the topical distribution of luliconazole [99].

Another investigation studied the antifungal effectiveness of liposome-loaded amphotericin B against *T. rubrum* and *T. interdigitale*. The study also explored the development of resistance through in vitro evaluation. The investigation included 29 archived clinical strains, comprising 13 *T. rubrum* and 16 *T. interdigitale* strains, which were identified using prior ITS1-ITS2 region sequencing. A thin-film hydration approach was employed to develop a liposomal formulation of amphotericin B. The CLSI M38-A2 broth microdilution technique was used to determine the minimum inhibitory concentration (MIC) of various antifungal agents, including itraconazole, efinaconazole, liposome-loaded amphotericin B, ciclopirox, and terbinafine. Comparing the liposome-loaded amphotericin B with individual liposomes and amphotericin B, the liposome-loaded version demonstrated a lower MIC. Importantly, the liposome-loaded amphotericin B did not induce drug resistance in any of the examined strains. This suggests that liposome-loaded amphotericin B could be an effective antifungal treatment for onychomycosis on the skin. In vitro testing did not indicate any resistance of the studied strains to liposome-loaded amphotericin B, suggesting that the use of this formulation is unlikely to induce drug resistance in dermatophyte species [100].

#### 5.1.2 Systemic medications (oral)

Itraconazole, ketoconazole, terbinafine, and fluconazole represent examples of antifungal agents that are presently employed in clinical practice. These medications are typically reserved for the treatment of severe or serious fungal infections, wherein topical antifungal therapies prove inadequate or inappropriate due to factors such as drug interactions, elevated costs, and potential adverse effects [101]. In addition to their high toxicity, dermatophytes have shown resistance to most available antifungal drugs, particularly against microbial biofilms. Therefore, it is crucial to search for new compounds with lower toxicity, novel mechanisms of action, and alternative targets. In this context, a recent study utilized nanostructured lipid systems (NLS) to load nonyl 3,4-dihydroxybenzoate, a potent agent against dermatophyte biofilms. The primary objective of the study was to enhance the solubility of the compound while maintaining its efficacy. The NLS formulation was prepared using a combination of soybean phosphatidylcholine (Epikuron® 200), polyoxyethylene lauryl ether (Brij®98), and cholesterol, along with PBS. The susceptibility of the NLS compound was assessed using the CLSI document M38-A2 (2008) guidelines. To evaluate toxicity, the compound was tested on HaCaT cell lines using the sulforhodamine B method, as well as in zebrafish and *Caenorhabditis elegans* models as alternative toxicity assays. Furthermore, the effectiveness of the compound was tested against mature biofilms of *T. mentagrophytes* and *T. rubrum*. The nonyl 3,4-dihydroxybenzoate loaded NLS and free NLS exhibited zeta potentials ranging from 1.46 to 4.63 mV, polydispersity indices (PDIs) ranging from 0.331 to 0.377, and sizes ranging from 137.8 to 167.9 nm. The synthesis of NLS of the microemulsion type was confirmed through polarized light microscopy studies. The minimum inhibitory concentration (MIC) of nonyl integrated into NLS ranged from 2 to 15.6 mg/L. The toxicity tests demonstrated more than 80% cell viability across all examined doses, as well as significant improvement in zebrafish and *Caenorhabditis elegans* survival. The anti-biofilm testing further confirmed the effectiveness of the incorporated compound. These findings hold significance in the search for novel antifungals as they enable systemic administration of the drug by improving effectiveness, bioavailability, reducing toxicity, and enhancing the solubility of non-polar agents [102].

**Fig. 5:**
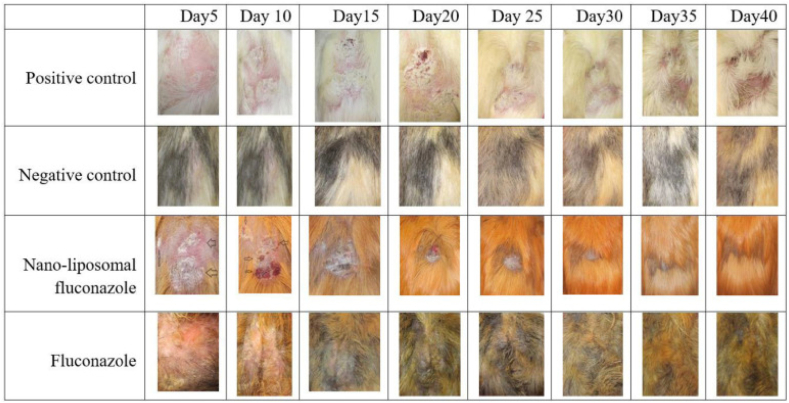
The recovery process was compared between the fluconazole 1% treatment group and the liposomal fluconazole treated group [103].

Dermatophytosis induced by *T. mentagrophytes* is a significant fungal infection affecting both animals and humans. Although antifungal agents like fluconazole and terbinafine are commonly used to treat dermatophytosis, the emergence of treatment resistance is a growing concern. To address this issue, the therapeutic efficacy of nanoformulations and conventional antifungal drugs was evaluated using a guinea pig model of dermatophytosis. *T. mentagrophytes* conidia were injected into the posterior dorsal region of 36 guinea pigs. The guinea pigs were divided into different groups and assessed clinically (lesion intensity and redness) as well as mycologically (culture and microscopy) until day 40 after inoculation. Therapy was initiated 5 days after inoculation and continued until day 40. On the first day of therapy, the groups treated with nano-drug versions of terbinafine and fluconazole exhibited a mean clinical lesion score of 3 (significant redness with substantial scaling), whereas the traditional forms of fluconazole and terbinafine scored 4. Between days 15 and 20, the nano-drug-treated groups demonstrated a reduction in lesion scores, which persisted until day 40. By day 40, all groups except the positive control group had a score of zero (refer to Fig. 5). Based on this research, nano-drugs appear to be more effective in managing dermatophytosis and hold potential as therapeutic options for the treatment of this condition [103].

Thyme essential oil, known for its high content of carvacrol and phenolic thymol, exhibits potent antibacterial, antiparasitic, and antifungal properties. To enhance their efficacy, protect against oxidation, and improve solubility, encapsulation techniques are utilized. A novel study was conducted to evaluate the antifungal activity of Zataria multiflora species encapsulated with nano-systems by loading Thymus essential oil (from Zataria multiflora species and Thymus diagenesis Celak) into liposomal vesicles. Liposomes loaded with Thymus essential oil were synthesized using the thin film hydration technique. The impact of cholesterol levels, Thymus species, and types of phospholipids on the entrapment efficiency was examined. The antifungal activity of liposomes loaded with Zataria multiflora species extract against *T. mentagrophytes* was assessed using the zone of inhibition, minimum inhibitory concentration, and lowest fungicidal concentration. The Thymus essential oil was successfully encapsulated in liposomes with an efficiency of over 80%. The optimized formulation included 3% polyethylene glycol, 90% soybean phosphatidylcholine phospholipid, 10% cholesterol, and 0.5 mg/mL Thymus essential oil. The nanoparticles exhibited spherical shape, anionic properties, and a size below 100 nm. No chemical interactions were observed between the essential oil and liposomes. The encapsulated compound remained chemically stable, and the essential oil maintained its properties. The medicinal-nano system of Zataria multiflora effectively inhibited *T. mentagrophytes*. The choice and quantity of phospholipids used in the ideal liposomal formulation containing Thymus essential oil influenced its characteristics, while the Thymus species did not have a significant impact. Encapsulation also enhanced the antifungal activity of Zataria multiflora essential oil [104].

Antifungal medications like fluconazole are effective in treating dermatophytosis. However, instances of medication resistance have been reported, necessitating the development of modern therapeutic approaches. In a recent study, the antifungal sensitivity of conventional fluconazole and nano fluconazole on dermatophyte isolates was investigated using the CLSI M38-A2 criteria. PCR sequencing methods were employed to identify dermatophyte species isolated from clinical cases of dermatophytosis. Liposomal fluconazole exhibited a zeta potential of −20.12 mV and a size of 88.9 nm. The encapsulation rate of fluconazole was 75%. Among the studied species, *T. interdigitale*, *E. floccosum*, and *T. rubrum* isolates demonstrated MIC50 values of 32, 8, and 16 μg/mL, respectively. In contrast, the corresponding concentrations of nano fluconazole for these three species were 8 μg/mL. Notably, conventional fluconazole exhibited higher MIC values than nano-fluconazole across all studied dermatophyte species. This suggests that nano-fluconazole might effectively inhibit dermatophyte growth at lower drug concentrations compared to fluconazole [50].

The incidence of fungal diseases caused by the Candida genus has been increasing in recent years, driven by factors such as the growing population of immunosuppressed individuals and the emergence of resistant strains. Liposomes, nanovesicles consisting of lipid bilayers, have been used to encapsulate various substances. In this study, the antifungal capability of bisabolol, a well-known sesquiterpene with established biological functions, was examined alone and in combination with fluconazole in liposomal form. Antifungal experiments were conducted using microdilution against standard strains of *C. krusei*, *C. tropicalis*, and *C. albicans* to determine the inhibitory concentrations (IC50) and evaluate microorganism survival. Morphological changes were assessed in micro-culture chambers based on the concentrations of liposomal bisabolol used in the experiments. The combination of liposomal bisabolol and fluconazole (FCZ) exhibited an IC50 value of 2.5 μg/mL against all tested strains, indicating a potentiation effect on their antifungal activity. Liposomal bisabolol was found to enhance the potency of fluconazole against *C. tropicalis* and *C. albicans* strains by reducing the concentration of fluconazole in the solution and completely suppressing fungal growth. Furthermore, a concentration of 1/8 of liposomal bisabolol prevented morphological changes in all tested isolates. The liposomal formulation demonstrated homogeneity, with vesicles exhibiting a surface charge potential of 34.2 mV and a size of 203.8 nm. These characteristics ensured the long-term stability of the nanosystem. Scanning microscopy confirmed the spherical shape of the vesicles. In summary, liposomal bisabolol, both alone and in combination with fluconazole, exhibited potent antifungal activity against Candida strains, providing a promising approach for combating Candida-related fungal diseases [105].

In summary, liposomal formulations have demonstrated significant potential as efficient delivery systems for antifungal medications. They enhance the effectiveness of the drugs, improve their ability to penetrate tissues, and minimize unwanted side effects. The use of liposomes holds potential for the treatment of dermatophyte infections, including superficial skin infections and onychomycosis, as well as Candida-related fungal diseases. Further research and development in this field can lead to novel and improved therapeutic options for fungal infections. Table 2 provides a summary of various liposomal formulations used for the management of dermatophyte infections, including their key findings and potential benefits.

**Table 2:** Summary of various liposomal formulations used for the management of dermatophyte infections, including their key findings and potential benefits.

Topic	Liposomal Formulation	Key Findings
**Topical Medications**	Liposomes loaded with griseofulvin incorporated into chitosan film	Enhanced drug permeation and faster flux rate observed in liposomal film formulation compared to liposomal formulations alone. Demonstrated antifungal effectiveness against *E. floccosum* and *M. gypseum* in vitro. Liposomal composite film showed potential for delivering griseofulvin to superficial fungal infections.
	TBF-HCl-loaded liposomes	TBF-HCl-loaded liposome film formulations demonstrated superior antifungal activity on fungal nails compared to other formulations. Higher drug permeation and cumulative drug permeation observed in liposome film formulations. Promising option for the treatment of fungal nail infections.
	Voriconazole-loaded liposomes	Liposomes showed higher drug accumulation in the skin and enhanced drug release, leading to higher drug levels in the skin compared to gel formulations. Liposomes demonstrated potential for limiting fungal growth against *T. rubrum*.
	Itraconazole-loaded liposomes	Optimized liposome formulation showed high percent drug entrapment, skin retention, and permeation. Liposome gel exhibited faster relief from infection compared to commercial topical and oral antifungal treatments in a Tinea pedis animal model.
	Liposome TH cream	Liposome TH cream showed higher drug release throughout the stratum corneum layers, providing a greater dose of the drug. Promising for the treatment of superficial mycoses.
	Elastic ethogel and lipo gel	Elastic ethogel and lipo gel formulations demonstrated nanometric vesicle size, high encapsulation efficiency, and enhanced antifungal activity against dermatophytes and Candida albicans. Improved skin deposition compared to a commercial formulation.
	Liposome-loaded amphotericin B	Liposome-loaded amphotericin B showed lower minimum inhibitory concentration (MIC) compared to individual liposomes and amphotericin B. No induction of drug resistance observed in dermatophyte strains. Effective antifungal treatment for onychomycosis on the skin.
**Systemic Medications (Oral)**	Liposomal fluconazole	Nano-fluconazole exhibited lower minimum inhibitory concentration (MIC) compared to conventional fluconazole against dermatophyte isolates. Potential for effectively inhibiting dermatophyte growth at lower drug concentrations.
	Liposomal of fluconazole and terbinafine	Nano-drug versions of fluconazole and terbinafine demonstrated a reduction in lesion scores and improved therapeutic efficacy compared to traditional forms in a guinea pig model of dermatophytosis.
	liposomal of fluconazole	Nanoformulations of fluconazole exhibited higher potency and lower minimum inhibitory concentration (MIC) compared to conventional fluconazole against dermatophyte species.
	Liposomal bisabolol	Liposomal bisabolol enhanced the antifungal potency of fluconazole against Candida strains, reducing the concentration of fluconazole needed for complete suppression of fungal growth. Promising approach for combating Candida-related fungal diseases.

## 6 Marketed products

There are some approved liposomal formulations with antifungal active pharmaceutical ingredients (API) in the United States and the European Union (EU). AmBisome® was the first liposomal formulation of amphotericin B that was developed to replace existing formulations by the Nexatar Company USA in 1990 [106]. Globally, several amphotericin liposomes are marketed, such as FungisomeTM and AnfogenTM. Many of their chemical components are the same as those in AmBisome® [107]. Econazole, fluconazole, clotrimazole, and other antifungal agents have been incorporated into liposomes for ex-vivo experiments [108]. Upon completion of these studies and clinical trials, we can expect more liposomal antifungal drugs to be available on the market shortly.

## 7 Conclusion, challenges and prospects

Advancements in nanotechnology within the medical field hold great potential for significant breakthroughs in disease prevention, diagnosis, and treatment. Nanoscience has revolutionized the approach to treating and diagnosing diseases and injuries, with various nanotechnology applications currently undergoing experimentation. These promising developments pave the way for a future in medicine that offers exciting opportunities to save lives globally. In the context of dermatophyte infections, recent studies have focused on the use of liposomal nanoformulations as an innovative approach to treatment. The field of nanoscience and nanomedicine has been introduced as crucial tools in addressing dermatophyte infections, highlighting the advancements made in this area. Previous research in this field serves as a foundation for further exploration and development. A comprehensive examination of dermatophytes, including their characteristics, diagnostic methods, and treatment options, is presented. This thorough investigation provides a comprehensive understanding of the nature of dermatophyte infections and the challenges associated with their treatment. Liposomes, with their unique structural properties, have emerged as a promising platform for delivering antifungal agents to effectively combat dermatophytes. Liposomes have shown remarkable capabilities in carrying both water-soluble and fat-soluble drugs. They can exist in various forms such as suspensions, semi-solid aerosols, and powders. With a high drug load capacity and minimal alteration of the drug carrier's characteristics, liposomes provide an efficient means of drug delivery. The physical entrapment of drugs within liposomes ensures that the drugs remain in their original position and are shielded from enzymatic degradation. Despite the many advantages of liposomes, there are challenges to overcome. Liposomes have a short half-life and are associated with high production costs, limiting their widespread use. Additionally, their slow drug release kinetics pose challenges in delivering antibiotics and may contribute to drug resistance. Penetration into the innermost tissue layers is hindered by biological barriers, and only a fraction of liposomes exhibit long-term stability. In recent decades, significant progress has been made in the development of liposomal nanoformulations. These advancements have led to improved drug stability, minimized adverse effects, enhanced effectiveness, and reduced toxicity. These developments have created a promising outlook for the future treatment of dermatophyte infections. It is anticipated that liposomal nanoformulations will play a significant role in overcoming the limitations associated with conventional treatments and offer effective solutions for managing dermatophyte infections. In conclusion, the integration of nanotechnology, specifically liposomal nanoformulations, into the field of dermatophyte treatment holds tremendous potential. The progress made in this area opens new avenues for improved drug delivery, enhanced therapeutic outcomes, and ultimately better patient care. Continued research and development in this field are crucial to unlock the full potential of liposomal nanoformulations and realize their impact on the treatment of dermatophyte infections.

## Ethics approval and consent to participate

This study with Reg. No. 400001182 was approved by the ethical committee of 10.13039/501100004621Kerman University of Medical Sciences. The Ethic approval Code is IR. KMU.REC.1401.103.

## Consent for publication

Not applicable.

## Author contribution statement

All authors listed have significantly contributed to the development and the writing of this article. </p>

## Data availability statement

No data was used for the research described in the article.

## Declaration of competing interest

The authors declare that they have no known competing financial interests or personal relationships that could have appeared to influence the work reported in this paper.
